# Thickness effect on optical behavior of monolithic zirconia

**DOI:** 10.6026/973206300213951

**Published:** 2025-10-31

**Authors:** Manisha Yersong, Akshay Bhargava, Rajiv Kumar Gupta, Bharti Dua1, P. Lakshmi Priyadarsini, Rushikesh Kaknale

**Affiliations:** 1 Department of Prosthodontics and Crown & Bridge, Santosh Dental College and Hospital, Santosh Nagar No. 1, Pratap Vihar, Ghaziabad, Uttar Pradesh, India; 2 Dean Dental, Santosh Dental College and Hospital, Santosh Nagar No. 1, Pratap Vihar, Ghaziabad, Uttar Pradesh, India

**Keywords:** Monolithic zirconia, thickness variation, translucency, color stability, spectrophotometry, veneered zirconia

## Abstract

Achieving optimal esthetics in all-ceramic restorations remains a challenge due to variations in translucency and color influenced by material thickness. Therefore, this study aimed to evaluate the effect of thickness on the optical properties of monolithic zirconia compared to veneered zirconia. Fifty zirconia discs were fabricated in varying thicknesses (0.5, 1.0, 1.5, and 2.0 mm) and analyzed for color and translucency using a spectrophotometer. Veneered zirconia exhibited the highest lightness, while 0.5 mm monolithic zirconia appeared darkest with clinically unacceptable ΔE values. The 1.0 mm monolithic zirconia showed the closest match to veneered zirconia with acceptable color differences. Overall, increasing thickness reduced translucency, and a 1.0 mm thickness offered the most esthetically favorable balance for clinical applications.

## Background:

The increasing esthetic expectations in restorative dentistry have led to extensive research on the optical performance of monolithic zirconia ceramics. Advances in material formulation and sintering technology have aimed to overcome the inherent opacity of conventional zirconia, promoting translucency comparable to glass-ceramics. Studies have demonstrated that modifications in yttria content, grain size and sintering parameters significantly influence the optical behavior of zirconia, particularly its light transmission and color stability [1, 2]. Alghazzawi [1] reported that long-term aging alters the crystalline phase composition and consequently the optical properties, emphasizing the need to balance esthetics with durability. Variations in translucency between multilayer and monolithic systems have also been linked to differences in layer composition and thickness. Alnassar [2] highlighted that surface treatments, coloring procedures, and fabrication conditions play crucial roles in determining the optical outcome. Huh *et al.* [3] investigated the influence of polishing procedures on the surface and optical characteristics of monolithic zirconia. The study aimed to determine how different surface treatments affect light transmission, translucency, and overall esthetic appearance. Understanding these effects is crucial for optimizing the finishing protocols of zirconia restorations to achieve natural and durable esthetics. The translucency of monolithic zirconia has been compared with lithium disilicate and other esthetic ceramics, with results indicating that while zirconia still exhibits lower translucency, optimized processing can yield clinically acceptable results [4, 6]. Arslan and Degirmenci [5] further showed that surface finishing and polishing influence the final perceived color, reinforcing the importance of post-processing procedures. Elsaka [7] evaluated newly developed multilayer zirconias and confirmed that structural gradient design enhances translucency without compromising strength. Meanwhile, Farzin *et al.* [8] found that external staining can modify optical properties and surface roughness, influencing light reflection. Harada *et al.* [9] compared various monolithic materials and concluded that zirconia's translucency depends mainly on crystalline phase distribution and particle size. Finally, Hatami *et al.* [10] observed that high-translucent zirconia, when used in reduced thicknesses, provides improved color matching, making it a viable option for conservative restorations. Collectively, these investigations [1-10] establish that monolithic zirconia's optical performance is multifactorial-governed by composition, thickness, sintering regime, and finishing. Understanding these interrelations is vital for achieving optimal esthetics without compromising mechanical integrity.

## Methodology:

An in-vitro experimental study was carried out in the Department of Prosthodontics, Santosh Dental College and Hospital, Ghaziabad, after obtaining ethical clearance from the Institutional Ethical Committee, Santosh Deemed to be University (Approval No. SU/2023/2490, dated 26/09/2023). A total of fifty zirconia disc specimens were fabricated and divided into two groups. Group 1 (control) consisted of ten veneered zirconia discs with a 0.5 mm zirconia core layered with 1.5 mm of porcelain. Group 2 (test) comprised forty monolithic zirconia discs, subdivided into four subgroups of ten discs each, with thicknesses of 0.5 mm, 1.0 mm, 1.5 mm and 2.0 mm, respectively. All discs were digitally designed in Meshmixer software and milled using a five-axis milling machine (DWX-50, Roland DG Corporation, Japan) to ensure uniformity. Following milling, specimens were sintered in a high-temperature furnace (T-crystal, Zhengzhou Brother Furnace Co., China) according to the manufacturer's recommendations and glazed with IPS e.max Ceram glaze materials (Ivoclar Vivadent, Liechtenstein). The optical properties of the specimens were evaluated using a PerkinElmer Lambda 1050+ spectrophotometer at the Central Research Facility, Sonipat (Research Extension of IIT Delhi). Measurements were recorded under D65 standard illumination and a 2° standard observer. Reflectance data in the visible spectrum (380-780 nm) was collected, converted to CIE XYZ tristimulus values and subsequently transformed into CIE Lab* coordinates following the CIELAB 1976 system. The color difference (ΔE) between veneered and monolithic zirconia samples was calculated using the standard formula. Statistical analysis was performed using one-way ANOVA to compare mean L*, a* and b* values across the groups, followed by post-hoc Tukey's test for pairwise comparisons, with a significance threshold set at p ≤ 0.05.

## Results:

This *in vitro* study assessed the optical properties of monolithic zirconia at different thicknesses (0.5 mm, 1.0 mm, 1.5 mm and 2.0 mm) in comparison to veneered zirconia (control). A total of 50 specimens were evaluated and their CIE Lab* values were measured using a spectrophotometer under standardized conditions. The results demonstrated that the control group (veneered zirconia) had the highest mean lightness (L* = 69.31), whereas the 0.5 mm monolithic zirconia group showed the lowest mean lightness (L* = 60.56), indicating a darker appearance. Among the monolithic groups, the 1.0 mm specimens exhibited optical properties closest to veneered zirconia, with minimal deviation in L*, a* and b* values. Color difference analysis (ΔE) revealed that the 1.0 mm group had the lowest mean ΔE (2.50), within the clinically acceptable threshold (<3.7), suggesting an excellent color match to veneered zirconia. In contrast, the 0.5 mm group showed the highest ΔE (9.67), indicating significant perceptible difference. The 1.5 mm and 2.0 mm groups also demonstrated moderate deviations (ΔE = 5.80 and 6.20, respectively), both statistically different from the control. Statistical analysis using one-way ANOVA confirmed significant differences across groups for L*, a* and b* values (p < 0.001). Tukey's post-hoc test showed that only the 1.0 mm group (Group 2B) was not significantly different from veneered zirconia (p > 0.05), whereas the other thicknesses differed significantly (p < 0.05) (Table 1 - see PDF).

## Discussion:

Optical characterization of zirconia remains an evolving field, as numerous variables influence its ability to transmit and scatter light. The interplay between microstructure, thickness, and fabrication parameters determines the final color and translucency perceived in clinical restorations. Ilie [11] provided one of the earliest systematic quantifications of light transmission through zirconia, demonstrating a clear inverse relationship between material thickness and transmittance. He also emphasized that curing conditions and illumination angles substantially alter the measurable translucency outcomes. Building on this, Kang [12] evaluated multilayer zirconia and reported that even minimal thickness variations, ranging from 0.5 mm to 1.5 mm, resulted in significant changes in ΔE values, indicating that precise thickness control is essential during restorative design. Kim [13] explored the effect of pre-coloring on the optical properties of monolithic zirconia. His investigation revealed that pigment infiltration before sintering slightly reduces translucency but enhances color uniformity across restorations. In a subsequent study, Kim [14] examined the influence of mechanical thickness reduction and found that although thinning improves light transmission, excessive reduction leads to perceptible color deviation and compromises esthetic harmony. Kontonasaki [15] provided an updated overview of zirconia's optical, wear, and clinical behaviors, concluding that achieving an optimal balance between translucency and mechanical performance remains critical for long-term success in anterior esthetics. Miura [16] examined the effects of sintering temperature on the translucency and color of zirconia. His findings indicated that increasing the sintering temperature up to an optimal point enhances translucency by promoting grain coalescence and reducing porosity. In a related investigation, Miura [17] assessed the impact of yttria concentration and sintering protocols, observing that higher yttria content improves light diffusion but may introduce cubic phase dominance, which can reduce strength. Pekkan [18] corroborated these findings by demonstrating that optimized sintering cycles lead to superior translucency and flexural strength, thereby validating the use of precisely controlled thermal profiles in laboratory practice. The study by Cho and Seol [19] demonstrated that introducing a yttria gradient in multi-layered monolithic zirconia enhances translucency and color blending while maintaining structural integrity. The graded design improved the balance between esthetics and strength by optimizing the cubic-to-tetragonal phase distribution. These findings highlight the potential of compositional engineering to achieve natural optical properties without compromising mechanical performance. Sulaiman [20] measured light irradiance through different zirconia thicknesses and found an exponential attenuation pattern as thickness increased. His work highlighted potential limitations in the polymerization of resin cements beneath thicker zirconia crowns, a consideration of clinical relevance for luting protocols. Tabatabaian [21] investigated the color characteristics of monolithic zirconia and emphasized that the final perceived shade depends on the interplay of restoration thickness, background substrate, and cement color. Later, Tabatabaian [22] evaluated high-translucency zirconia and observed that thinner specimens exhibited improved color matching, suggesting that clinical success depends on harmonizing optical and structural requirements.

Tavangar [23] studied the effects of bleaching agents on externally stained zirconia surfaces and reported that such chemical interventions can diminish both color stability and surface integrity. The findings underscore the need for caution when performing whitening treatments in patients with zirconia restorations. Toksoy [24] provided a comprehensive overview of the evolution of zirconia generations, documenting a gradual transition from traditional 3Y-TZP to modern 5Y-PSZ formulations. His study confirmed that a higher cubic phase fraction enhances translucency but compromises toughness, highlighting the ongoing challenge of optimizing both optical and mechanical performance. Zhang [25] expanded on this principle by analyzing novel zirconia materials engineered with refined microstructures that simultaneously enhance translucency and resistance to low-temperature degradation, thereby offering promising directions for future dental ceramics. The collective evidence demonstrates that translucency and color behavior in zirconia are not governed solely by thickness but result from complex interactions among chemical composition, sintering dynamics, and surface finishing. Ilie [11] and Kang [12] established the foundational relationship between material thickness and light behavior, while Kim [13, 14] and Kontonasaki [15] expanded the discussion to include color control and long-term esthetic stability. The microstructural insights from Miura [16, 17] and Pekkan [18] reinforced the importance of optimizing sintering parameters to achieve uniform grain growth and minimize scattering interfaces. Clinical research, such as that by Sarikaya [19] and Sulaiman [20], has translated these laboratory findings into practical implications for veneer and crown fabrication, ensuring predictable shade outcomes and appropriate curing depth for luting agents. Similarly, investigations by Tabatabaian [21, 22] and Tavangar [23] remind clinicians that external factors-substrate color, cement shade, and even post-treatment bleaching-can significantly influence esthetic results. Toksoy [24] and Zhang [25] collectively present a forward-looking perspective, showing that the latest zirconia systems integrate microstructural refinements to enhance translucency while maintaining mechanical durability suitable for both posterior and anterior use. In summary, the optical performance of monolithic zirconia is multifactorial. Advances in material formulation, sintering control, and surface treatment have elevated zirconia from a primarily structural core material to one capable of delivering high esthetic quality. Ongoing developments in high-yttria and gradient ceramics show promise for further improving light transmission and shade fidelity without sacrificing mechanical strength. Continued research integrating optical, mechanical, and biological parameters will help establish standardized protocols for material selection, ensuring both visual harmony and long-term clinical reliability across restorative applications.

## Conclusion:

The optical properties of monolithic zirconia were found to be highly dependent on material thickness. A 1.0 mm thickness demonstrated color and translucency values closest to veneered zirconia, providing the most esthetically balanced outcome within clinically acceptable limits. Therefore, 1.0 mm monolithic zirconia may be considered optimal for achieving natural esthetics while maintaining sufficient masking ability in clinical restorations.

## Clinical message:

The optical performance of monolithic zirconia is highly thickness-dependent. A 1.0 mm thickness provides the best balance between translucency and masking ability, achieving esthetic outcomes comparable to veneered zirconia while avoiding veneer-related failures. Ultra-thin restorations (<1.0 mm) risk esthetic compromise due to substrate influence, whereas overly thick restorations (>1.5 mm) may appear opaque. Clinicians should carefully select restoration thickness to optimize both function and esthetics.

## Learning point:

To assess the changes in optical properties of monolithic zirconia as affected by the thickness of the material.
